# Characterization of a Truncated Metabotropic Glutamate Receptor in a Primitive Metazoan, the Parasitic Flatworm *Schistosoma mansoni*

**DOI:** 10.1371/journal.pone.0027119

**Published:** 2011-11-01

**Authors:** Amira Taman, Paula Ribeiro

**Affiliations:** Institute of Parasitology, McGill University, Macdonald Campus, Sainte Anne de Bellevue, Quebec, Canada; Royal Tropical Institute, Netherlands

## Abstract

A novel glutamate-binding protein was identified in *Schistosoma mansoni*. The protein (SmGBP) is related to metabotropic glutamate receptors from other species and has a predicted glutamate binding site located within a Venus Flytrap module but it lacks the heptahelical transmembrane segment that normally characterizes these receptors. The SmGBP cDNA was cloned, verified by 5′ and 3′ Rapid Amplification of cDNA Ends (RACE) and shown to be polyadenylated at the 3′end, suggesting the transcript is full-length. The cloned cDNA was subsequently expressed in bacteria and shown to encode a functional glutamate-binding protein. Other studies, using a specific peptide antibody, determined that SmGBP exists in two forms, a monomer of the expected size and a stable but non-covalent dimer. The monomer and dimer are both present in the membrane fraction of *S. mansoni* and are resistant to extraction with high-salt, alkaline pH and urea, suggesting SmGBP is either an integral membrane protein or a peripheral protein that is tightly associated with the membrane. Surface biotinylation experiments combined with western blot analyses and confocal immunolocalization revealed that SmGBP localized to the surface membranes of adult male schistosomes, especially the dorsal tubercles. In contrast, we detected little or no expression of SmGBP either in the females or larval stages. A comparative quantitative PCR analysis confirmed that the level of SmGBP expression is several-fold higher in male worms than cercariae, and it is barely detectable in adult females. Together, the results identify SmGBP as a new type of schistosome glutamate receptor that is both gender- and stage-specific. The high-level expression of this protein in the male tubercles suggests a possible role in host-parasite interaction.

## Introduction

The parasitic flatworm, *Schistosoma mansoni* is the major cause of human schistosomiasis, a disease that afflicts nearly 200 million people worldwide [1]. *S. mansoni* has a complex life cycle that requires two hosts, a freshwater snail of the genus *Biomphalaria* and the definitive mammalian (human) host. Humans become infected when free-living freshwater larva of *S. mansoni* (cercariae) penetrate the skin and are quickly transformed into a parasitic larval stage (schistosomula). The newly transformed larvae then enter the circulation and undergo a complex migration through the lungs and heart towards the hepatoportal system, where they continue to develop to adult male and female worms and egg production begins. The pathology associated with schistosomiasis is due mainly to granulomatous inflammatory responses induced by large numbers of eggs that become lodged in host tissues. The arsenal of drugs available for treatment of schistosomiasis is very limited. Praziquantel is the only drug available in most parts of the world and there are growing concerns about the prospect of drug resistance. There is an urgent need to learn more about the basic biology of this organism and to identify new molecular targets for drug development.

The nervous system of schistosomes is an attractive target for chemotherapeutic intervention. *S. mansoni* has a well developed central nervous system (CNS) and an extensive peripheral system of minor nerve fibers and plexuses that coordinate all major activities of the parasite [2]. Of particular interest as potential drug targets are components of the nervous system that control neuromuscular signaling related to movement, host attachment and migration, as well as sensory neurons located at the surface that may be involved in host-parasite interactions. A number of neurotransmitter systems and receptors have been identified in *S. mansoni* [2]–[4]. Here we focus our attention on L-glutamate, an important neurotransmitter of many vertebrate and invertebrate phyla. Glutamate-containing neurons have been identified in a variety of flatworm species [5]–[8], including *S. mansoni* [9], and there is evidence implicating glutamate in the regulation of neuromuscular activity in these worms. For example, glutamate was shown to stimulate muscle contraction when applied onto isolated muscle fibers of *S. mansoni* [10] and muscle strips of the tapeworm, *Hymenolepis diminuta* [11]. Moreover, treatment of cultured schistosomes with glutamate agonists produced strong body wall contractions and hyperkinesis [12], suggesting a probable role in the coordination of the somatic muscles and movement. The mechanisms responsible for these effects are largely unknown. There are several predicted glutamate receptors encoded in the genomes of *S. mansoni* [13] and the related schistosome species, *S. japonicum* [14] but most of these receptors have yet to be characterized at the molecular level.

In other organisms, glutamate exerts its effects by interacting with multiple types of cell-surface receptors, both ionotropic gated channels and metabotropic glutamate receptors (mGluRs) [15]. The mGluRs belong to the superfamily of G protein-coupled receptors (GPCR) and share a common heptahelical transmembrane (7-TM) topology. Vertebrates have eight mGluRs, which are classified according to three major groups based on sequence homology and mechanisms of signal transduction. Group I receptors (mGluR1 and mGluR5) are coupled to Gq/11 proteins and signal through changes in intracellular calcium and the inositol phospholipid pathway. In contrast, Group II (mGluR2 and mGluR3) and Group III receptors (mGluR4, mGluR6, mGluR7 and mGluR8) bind to Gi/o proteins and signal primarily through inhibition of adenylate cyclase and a decrease in cellular cAMP [15]. Besides vertebrates, mGluRs have also been identified in several invertebrate species, particularly insects and nematodes. Group I, II and III mGluR homologues have been described in *Drosophila* and *Caenorhabditis elegans* [16], [17] suggesting these major groups of receptors diverged early in evolution. In addition, insects have a type of mGluR (named mGluR X) that is distantly related to Groups II/III receptors and may be unique to invertebrates [18].

All vertebrate and invertebrate mGluRs cloned to date belong to a subset of GPCRs (Family C) that also includes metabotropic γ-aminobutyric acid (GABA) receptors, calcium-sensing, taste and pheromone receptors, among others. Family C GPCRs have a distinctive modular structure, consisting of a large N-terminal extracellular domain (ECD), followed by the signature 7-TM segment and an intracellular C-terminal region of variable length. The ECD of mGluRs contains the glutamate binding site located within a Venus Flytrap module and is connected to the 7-TM region by a short cysteine-rich linker [19]. The ECD is structurally related to bacterial periplasmic binding proteins (PBP) and they share a common mechanism of ligand binding [20]. It has been suggested that the modular structure of mGluRs evolved from fusion of two genes encoding a bacterial –like periplasmic binding protein and an ancestral heptahelical (7-TM) membrane receptor [21]. mGluRs may function as monomers, dimers and larger oligomeric species. Dimerization, in particular, has been well demonstrated among these receptors and plays an important role in ligand binding and activation [22]–[24].

The *S. mansoni* genome encodes at least three sequences that share homology with mGluRs from other species [13]. We have previously reported that one of these sequences, named SmGluR, encodes a functional glutamate receptor, which is expressed in part in the worm's central nervous system [25]. In this study we describe the second and most unusual of these predicted receptors. The *S. mansoni* glutamate-binding protein (SmGBP) reported here resembles the ECD of a metabotropic glutamate receptor but lacks the remaining domains, including the signature 7-TM region. Genes encoding similarly truncated receptors were found in the *S. japonicum* genome [14] and the partially annotated genome of the planarian, *Schmidtea mediterranea* [26] but are not known to occur in other metazoans. We conclude that SmGBP represents a new type of glutamate receptor, one that may be unique to flatworms. Here we provide a first investigation of this novel schistosome receptor through biochemical analyses, functional expression studies and confocal immunolocalization.

## Materials and Methods

### Parasites

A Puerto Rican strain of *S. mansoni* was used in all the experiments. *Biomphalaria glabrata* snails infected with *S. mansoni* were obtained from Dr. F. Lewis, Biomedical Research Institute (Bethesda, MD). Snails were induced to shed after light exposure and cercariae were mechanically transformed into schistosomula, as described [27], [28]. Adult worms were obtained 6–8 weeks post-infection of 28 day-old CD1 female mice by portal perfusion [27]. When required, males and females were separated by incubating freshly recovered worms in Dulbecco's Modified Eagle Medium (DMEM) (Invitrogen) for 4 h at room temperature. All animal procedures were approved by a McGill University Facility Animal Care Committee (FACC) and were performed in accordance to FACC animal protocol # 3346.

### Cloning of SmGBP

An expressed sequence tag (EST) was first identified in the S. *mansoni* EST database as a partial mGluR (Sm10811) [29]. The missing 5′ and 3′ends were obtained by RACE (Rapid Amplification of cDNA Ends) procedures, using commercial kits (Invitrogen). For the 3′RACE, total RNA was extracted from adult *S. mansoni* with TRIzol® reagent (Invitrogen) and reverse-transcribed using the oligo-dT anchor primer supplied by the kit. The resulting cDNA was used in a PCR with a sense gene-specific primer (5′-TGGGTGGGGTAGAGAAAATG-3′) and the kit antisense primer targeting the anchored sequence. Nested PCR was done using an internal gene-specific primer (5′-CATCATTACCTTCATCGTGC-3′) and the same reverse anchor primer. For 5′RACE, total RNA from adult *S. mansoni* was reverse-transcribed using a gene-specific primer (5′-GTAGTACCCAAAGTTAAACCTG-3′) and an anchor sequence was added to the 5′end, according to the kit protocol (5′RACE, Invitrogen). The resulting cDNA was used as a template for PCR, using a forward primer targeting the 5′ end anchored sequence (supplied by the kit) and a gene-specific reverse primer (5′-CCAAGCATTATATCACCTG-3′). A second round of PCR was done using a gene-specific internal primer (5′-TATAATAGTAACCAAATGAAAC-3′) as antisense and the same anchor primer as sense. The 3′ and 5′ RACE products were cloned into pGEM®-T Easy Vector (Promega) and confirmed by DNA sequencing of multiple clones. For expression of recombinant SmGBP, the predicted coding sequence was amplified directly from oligo-dT reverse-transcribed total RNA extracted from adult *S. mansoni.* The open reading frame of SmGBP has two potential start codons that are 15 bp apart (positions 184 and 199 of full length cDNA). To clone the coding sequence, we amplified a 1452bp cDNA from oligo-dT reverse-transcribed adult *S. mansoni* RNA, using primers that targeted the second start codon at position 199 (forward primer: 5′-ATGTTGATTACTTTAATGATTATATTATGG-3′) and the predicted stop codon at position 1650 (reverse primer: 5′-CTAACGTATAAACTCACCAGTAAATG-3′). Efforts to amplify a product from the first predicted start codon were unsuccessful, despite repeated attempts. The final product was cloned into pJET1.2/blunt vector (Fermentas) and confirmed by sequencing of two separate clones.

### Antibody production

A polyclonal anti-SmGBP antibody was purchased from Antagene, Inc. (Sunnyvale, CA, USA). The antibody was produced in rabbits against two synthetic peptides corresponding to positions 53-67 (GVTTCESINPERGIQ) and positions 461-475 (CLEKMLSYPGTAFYQ) of SmGBP. Each of the peptides was conjugated to keyhole limpet haemocyanin (KLH) as a carrier. Peptide sequences were examined by BLAST analysis against the Schistosome Genome Database as well as the general protein database at The National Center for Biotechnology Information (NCBI) (http://www.ncbi.nlm.nih.gov/) to insure specificity. Antisera were tested first against the two peptides by an ELISA test and shown to be of high titer. The antiserum was subsequently preadsorbed to KLH (Sigma) and then affinity-purified using the MicroLink™ Peptide Coupling kit (Pierce), as described previously [30].

### Western blotting and immunoprecipitation of native SmGBP

A membrane protein fraction was prepared from adult *S. mansoni*, using the ProteoExtract®Native Membrane Protein Extraction Kit (Calbiochem) as described in the kit protocol. For western blotting, equal amounts of membrane proteins and the remainder, predominantly soluble proteins (≈ 10 µg) were resolved on a precast 4-12% gradient Tris-Glycine SDS-PAGE gel (Invitrogen), transferred onto PVDF filters and incubated overnight with peptide-purified anti-SmGBP antibody (dilution 1∶2000). The blots were washed and incubated with Horseradish Peroxidase (HRP)-conjugated goat anti-rabbit antibody (Calbiochem) (dilution 1∶20,000) and western positive bands were detected using standard procedures. Immunoprecipitation (IP) was performed using the Seize®Primary Immunoprecipitation kit (Pierce). Briefly, IP affinity columns were prepared by coupling 200 µg of anti-SmGBP antibody to Aminolink® Plus gel overnight at 4°C and then washed extensively. Solubilized *S. mansoni* membrane proteins were prepared with the Protein Extraction Kit described above and aliquots containing 50 µg of protein were loaded onto the IP column for 4 hours at room temperature. The column was washed extensively and the bound protein was eluted under acidic (pH 2.8) conditions, as described in the kit protocol. The eluted protein was immediately neutralized to pH 7.4 by addition of 50 mM Tris-HCl, pH 9.5. The acidic and neutralized samples were both tested by western blot analysis with affinity-purified anti-SmGBP antibody, as described above. In some experiments, the neutralized protein samples were incubated with 0.1 M dithiotheritol (DTT), 1% triton X-100 or 6 M urea at 37°C for 30 min prior to western blot analysis.

### Sequential extraction of *S. mansoni* proteins

To test whether SmGBP is a soluble or membrane protein, we performed sequential extractions with high salt, alkaline pH and finally urea [31]. Adult *S. mansoni* (about 60 mg wet weight) were homogenized in buffer containing 2 M NaCl, 10 mM HEPES-NaOH, pH 7.4, 1 mM EDTA (ethylene diaminetetraacetic acid) and then centrifuged at 125,000g for 15 min at 4°C. The resulting pellet was resuspended in 0.1 M Na_2_CO_3_, 1 mM EDTA, pH 11.3, incubated on ice for 30 min and the insoluble material was collected by centrifugation as before. The pellet was resuspended in 10 mM HEPES-NaOH pH 7.4, containing 4 M urea, 100 mM NaCl and 1 mM EDTA. The final pellet containing membranes was obtained by centrifugation for 20 min, 4°C at 125,000g. A protease inhibitor cocktail (Sigma Protease Inhibitor Cocktail) was used throughout the entire experiment (added at 1∶100 final concentration). Aliquots of all supernatant fractions and the final resuspended pellet were subjected to SDS-PAGE on a 4-12% Tris-Glycine gel (Invitrogen) and blotted onto a PVDF membrane. Western blot was done using affinity-purified anti-SmGBP antibody (dilution 1∶2000) and anti-rabbit Horseradish Peroxidase-conjugated secondary antibody (1∶20,000 dilution).

### Expression of SmGBP in mammalian cells

The 1452bp coding sequence of SmGBP was ligated between the *SalI* and *NotI* restriction sites of mammalian expression vector, pCI-neo, and the resulting The pCIneo-SmGBP plasmid was used for transient transfection of HEK-293 cells, using Fugene6 (Roche), according to the manufacturer's recommendations. Protein expression in the transfected cells was monitored by *in situ* immunofluorescence, as described previously [32], using affinity-purified anti-SmGBP antibody. The soluble and membrane fractions of transfected HEK-293 cells or mock transfected controls were extracted using the ProteoExtract®Native Membrane Protein Extraction Kit (Calbiochem), as above, and were tested for the presence of SmGBP by western blot analysis, using the same antibody. IP studies of transfected HEK-293 cells were performed exactly as described for the native protein, except that the recombinant SmGBP was found to be soluble in the mammalian cell environment and therefore was isolated from the cytosolic (rather than membrane) protein fraction. Aliquots of soluble proteins from SmGBP-expressing HEK-293 cells or mock-transfected controls were loaded onto an anti-SmGBP IP column and the protein was eluted, neutralized and probed with affinity-purified anti-SmGBP antibody, as described above.

### Immunolocalization of SmGBP in *S. mansoni*

For immunofluorescence confocal microscopy experiments, adult *S. mansoni* and larvae were prepared as previously described [25], [30]. Animals were incubated with peptide-purified anti-SmGPB antibody (dilution 1∶150) for 3-4 days at 4 °C with gentle agitation. Animals were washed overnight and then treated with Fluorescein Isocyanate (FITC)-conjugated goat anti-rabbit IgG antibody (Chemicon) (dilution 1∶ 1000) for 4 days at 4 °C. As a counterstain, we added 200 µg/ml of phalloidin-tetramethyl rhodamine isothiocyanate (TRITC) for the last three days of incubation with the secondary antibody. The samples were examined using a BIO-RAD RADIANCE 2100 confocal laser scanning microscope equipped with a Nikon E800 fluorescence microscope for confocal image acquisition and the LASERSHARP 2000 analyzing software package. Controls used in these studies include (1) replacement of the primary antibody with pre-immune serum (2) omission of the primary antibody (3) pre-adsorption with an excess of the combined SmGBP peptide antigens (0.25 mg/ml of each peptide).

### Surface biotinylation of live *S. mansoni*

*S. mansoni* surface labeling was done according to the method of Braschi and Wilson [33]. Freshly recovered adult worms were washed with Hanks' balanced salt solution (HBSS) (Invitrogen) and examined first under the microscope. Damaged worms were removed and were not used in the experiment. The worms were incubated with EZ-Link® sulfo-NHS-LC-Biotin (Pierce) (1.5 mg/ml prepared in HBSS) with gentle agitation at 4°C for 30 min. The reaction was terminated by washing the worms in RPMI 1640 (Invitrogen). To verify the labeling and integrity of the schistosome surface membrane, freshly labeled worms were incubated with Pierce® Streptavidin Fluorescein conjugated (1∶100 dilution) for 30 min at room temperature with end-over-end rotation. The worms were washed with RPMI 1640 and examined by confocal microscopy as above. For further analysis, we prepared a solubilized membrane fraction from biotin-labeled worms, using the same ProteoExtract®Native Membrane Protein Extraction Kit described earlier (Calbiochem). Biotinylated membrane proteins were subsequently purified with Pierce® Streptavidin Agarose Resin, according to the manufacturer's recommendations. Briefly, aliquots of membrane proteins (40 µg) were incubated with Streptavidin resin for 1 hour at room temperature, followed by centrifugation at 500g for 1 min at 4°C to remove unbound proteins. The resin was then washed five times with PBS (phosphate-buffered saline) and the bound biotinylated proteins were recovered by heating the sample in 1x SDS-PAGE sample buffer for 5 min at 90°C. Aliquots of total biotin-labeled membrane proteins, unbound proteins and streptavidin-purified (recovered) proteins were resolved by SDS-PAGE, blotted onto a PVDF membrane and probed both with streptavidin-horseradish peroxidase conjugate (Amersham) and anti-SmGBP antibody.

### Real-time quantitative PCR (qPCR) analyses

Total RNA was extracted from *S. mansoni* cercariae, adult males and females, using the RNeasy kit (Qiagen). The RNA was quantified with a Nanodrop ND1000 spectrophotometer (Wilmington, USA) and equal amounts of RNA from each stage (approximately 100 ng) were used for oligo-dT reverse-transcription, using M-MLV reverse transcriptase (Invitrogen), according to standard protocols. The real-time qPCR reaction was done with the Platinum SYBR Green qPCR SuperMix-UDG kit (Invitrogen), according to the kit protocol in a Rotor-Gene RG3000 (Corbett Research) real-time PCR cycler. The primers for qPCR were designed so as to amplify a SmGBP product of 127 bp (forward: 5′- TAACTGGACTTATGTTTCC-3′ and reverse: 5′- ATTTCCTCTAATCACTTCTC-3′) or a 206 bp fragment of *S. mansoni* glyceraldehyde-3-phosphate dehydrogenase (GAPDH; Accession# M92359; forward: 5′-GTTGATCTGACATGTAGGTTAG-3′; reverse: 5′-ACTAATTTCACGAAGTTGTTG-3′). GAPDH was used as a housekeeping gene for data normalization. The PCR cycling conditions were as follows: 50 °C/2 min, 95 °C/2 min followed by 50 cycles of 94 °C/15 s, 50 °C/30 s and 72 °C/30 s. Non-template and minus reverse-transcriptase controls were included in every experiment and the specificity of the PCR products was tested by melting curve analysis. Quantification of relative differences in expression was done by using the comparative ΔΔC_t_ method [34].

### Bacterial expression and purification of SmGBP

For functional studies in *E. coli*, the 1452bp coding sequence of SmGBP was amplified by PCR using primers designed to add *BamHI* and *XhoI* restriction sites at the 5′- and 3′-ends, respectively. The terminal stop codon was removed to add a six histidine tag at the C-terminal end of the expressed protein. The amplified sequence was then subcloned between the *BamHI* and *XhoI* sites of the pET prokaryotic expression vector, pET22b (Novagen). This vector carries a *pelB* signal sequence for periplasmic expression of cloned proteins. The resulting pET22b-SmGBP construct was confirmed by sequencing and then used to transform *Escherichia coli* host strain BL21 (DE3)pLysS (Novagen). The transformed *E. coli* cells were grown in LB (Luria-Bertani) liquid media supplemented with ampicillin and chloramphenicol at 37°C until the OD_600_ value reached 0.6-1 and the culture was then induced with 1 mM IPTG (Isopropyl β-D-thiogalactopyranoside) for 3 hours at 30°C. The periplasmic protein fraction containing recombinant SmGBP was isolated by osmotic shock, as described previously [35]. The cells were washed once with cold PBS then subjected to osmotic shock by incubation in 5 mM Tris–HCl, 20% sucrose, pH 8 for 10 min at room temperature with occasional vortexing and finally pelleted at 4°C. The cell pellet was resuspended in 10 ml ice-cold 5 mM MgSO_4_ containing a cocktail of protease inhibitors (Sigma Protease Inhibitor Cocktail, 1∶100 final concentration) and the cell suspension was mixed gently for 10 min on ice to release the periplasmic proteins. The remaining cell material was removed by centrifugation (10,000g for 10 min at 4°C) and aliquots of the supernatant containing recombinant oligohistidine-tagged SmGBP (SmGBP-His) were used directly for protein purification. The protein was purified by immobilized-nickel affinity chromatography, using His•Bind Resin and Buffer Kit from Novagen (EMD Biosciences, US), according to the kit protocol. The purified protein was immediately concentrated by ultrafiltration with a Centricon-10 microconcentrator (Amicon) and washed twice with storage buffer (0.2 M NaCl, 50 mM Tris-HCl and 10% glycerol, pH 7.2). The purification was verified by SDS-PAGE on a 4-12% Tris-Glycine gradient gel (Invitrogen) and western blotting with affinity - purified anti-SmGBP antibody. Aliquots of the purified SmGBP-His protein (final concentration 1 mg/ml) were frozen at -80°C in the same storage buffer until use.

### Ligand-binding assays

[^3^H]glutamate binding assays were performed as described previously [36]. Briefly, 5 µl (5 µg) of purified SmGBP-His were incubated with 1 µl of [^3^H]glutamate (specific activity 49.6 Ci/mmol; 1 mCi/ml) (Perkin Elmer-Canada) in 150 µl of binding buffer (40 mM HEPES, pH 7.5 and 2.5 mM CaCl_2_) in the absence or presence of different concentrations (0.1 nM to 100 µM) of unlabelled glutamate for 1 h at 4°C. The bound ligand was recovered by the polyethylene glycol (PEG) precipitation method [36]. 6 kDa PEG (Sigma) and γ-globulin (Sigma) were added to final concentrations of 15% and 3 mg/ml, respectively, and the samples were centrifuged for 15 min at 4°C. The precipitated material was washed twice, each for 10 min with binding buffer containing 8% 6 kDa PEG. The pellet was resuspended in 1ml of water and added to 5 ml of scintillation fluid (Universol™, ICNBiomedicals, CA). Radioactivity was measured using a 1414 WinSpectral™, Wallac scintillation counter. The same protocol was repeated in competition studies with glutamine, aspartate (Sigma) and various mGluR agonists and antagonists, including ((S)-3,5-DHPG, (S)-3,5-Dihydroxyphenylglycine; DCG IV, (2S,2′R,3′R)-2-(2′,3′-Dicarboxycyclopropyl)glycine); L-AP4, L-(+)-2-Amino-4-phosphonobutyric acid; (S)-MCPG, (S)-a-Methyl-4-carboxyphenylglycine; LY 341495, (2S)-2-Amino-2-[(1S,2S)-2-carboxycycloprop-1-yl]-3-(xan th-9-yl) propanoic acid), each at a final concentration of 10 mM. Neurochemicals were purchased from Tocris Bioscience, USA.

### Other methods

To test the specificity of the anti-SmGBP antibody, indirect ELISA was performed in 96-well plates coated with SmGBP peptides (1 µg/well) and a serial dilution (1∶ 250,000-1∶50) of rabbit anti-SmGBP antiserum or pre-immune serum, followed by incubation with a HRP-labeled goat anti-rabbit IgG (dilution 1∶1000) antibody, according to the standard protocols. Protein concentration was determined by the Lowry method, using a commercial kit from BioRad Canada. Data analysis was done with the Prism software package for the Macintosh version 3.0a (Graphpad Software, San Diego, CA, USA). ClustalW protein sequence alignments and the construction of phylogenetic trees were performed with MacVector 7.1.1 (Accelrys, Inc.). Trees were built according to the Neighbour-Joining method using the Best Tree mode available in MacVector and were verified by bootstrap analysis with 1000 iterations. The homology model of SmGBP was generated from a structural alignment with the crystal structure of rat mGluR 3 (Protein Data Bank (PDB) accession # 2e4u), using the modeling tools available at MODBASE [37] and was visualized with FirstGlance in Jmol version 1.45. Statistical comparisons were done with Student *t*-tests or a one-way analysis of variance (ANOVA) followed by a Tukey's pairwise analysis, as required. A *P* <0.05 was considered statistically significant.

## Results

### Cloning of SmGBP

A search of the *S. mansoni* EST database (http://bioinfo.iq.usp.br/schisto/) [29] identified a sequence (886bp) that resembled mGluRs from other species. This sequence was first amplified from oligo-dT reverse-transcribed adult *S. mansoni* cDNA. The missing 5′ and 3′ ends were subsequently obtained by RACE and verified by DNA sequencing of multiple clones. The complete cDNA has (1863 bp), including a stretch of 5′untranslated sequence (183 bp) followed by a large continuous open reading frame (ORF) of 1467 bp and a segment of 3′untranslated region (213 bp), which contains a polyA^+^ tail (Accession # HM483390). Following completion of the *S. mansoni* genome sequencing project, the predicted genomic sequence for SmGBP was annotated and designated Smp_052660 (CAZ32804.1) [13]. The genomic sequence is identical to that of the cloned cDNA, except for five nucleotide substitutions that result in amino acid changes at positions 21, 52, 84, 403 and 477 of the SmGBP protein sequence.

### Sequence analysis

**Figure 1:**
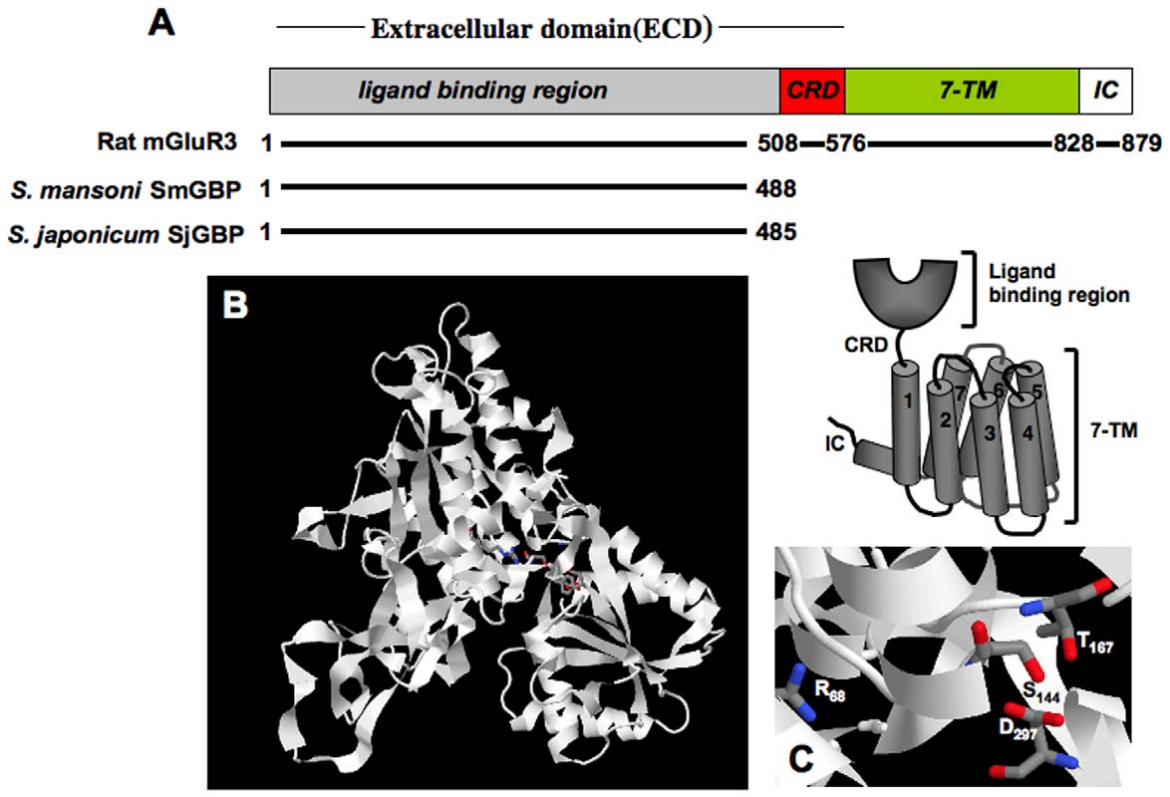
SmGBP is a C-terminally truncated metabotropic glutamate receptor. (A) Predicted structure of *Schistosoma mansoni* SmGBP (Accession # HM483390) and a closely related *Schistosoma japonicum* homologue SjGBP (Sjp 0088520.1) compared to the crystal structure of rat mGluR3 (group II subtype 3) receptor (2e4u). The cartoon illustrates the expected domain architecture of metabotropic glutamate receptors (mGluR). The extracellular binding domain (ECD) includes a conserved Venus Flytrap module, which contains the glutamate binding region and is linked to the heptahelical membrane-anchoring segment (7-TM) by a cysteine-rich domain (CRD). The 7-TM segment is followed by an intracellular C-terminal domain (IC) of variable length. The amino acid positions of the various domains in mGluR3 are marked. Compared to the rat receptor, the schistosome SmGBP and SjGBP sequences contain the ligand binding region but lack the remaining cysteine-rich, membrane spanning and intracellular domains. (B) Hypothetical homology model of SmGBP showing the characteristic bilobed Venus Flytrap architecture of the mGluR ligand binding domain. The model was generated from an alignment with the crystal structure of rat mGluR3 (2e4u) using the modeling tools available at MODBASE [37]. (C) Predicted ligand binding residues of SmGBP were identified by comparison with known glutamate binding sites of mGluR3 [38]. Binding sites that are conserved in SmGBP include (*corresponding mGluR3 position*): Arg_68_ (*Arg_68_*), Ser_144_ (*Ser_151_*), Thr_167_ (*Thr_174_*), Tyr_215_ (*Tyr_222_*) and Asp_297_ (*Asp_301_*).

The SmGBP cDNA has two potential start codons that are 15 bp apart (positions 184 and 199 of full length cDNA). The longest open reading frame (1467 bp) encodes a protein of 488 amino acid residues and a predicted molecular weight of 55 kDa. Blastp homology searches combined with multisequence alignments determined that SmGBP is closely related to the extracellular ligand binding region of metabotropic glutamate receptors but, surprisingly, lacks the remaining domains that normally characterize these receptors. The cysteine-rich linker (CRD), the 7-TM transmembrane segment and the C-terminal intracellular domain are all missing in the schistosome protein (Fig. 1A). These regions are also absent in the current annotation of the SmGBP genomic sequence available at NCBI (Smp_052660). As noted above, the cDNA was thoroughly verified by RACE analysis and sequencing of multiple clones, with particular emphasis on the 3′ end. We found no other forms of SmGBP that contained the missing sequence. Moreover, SmGBP was found to be polyadenylated at the 3′ end as would be expected of a mature, properly processed transcript. Thus we conclude that SmGBP encodes a C-terminally truncated mGluR. Further analysis of the primary protein sequence identified a hydrophobic peptide at the N-terminus that could be a signal peptide or possibly a single transmembrane domain (position 10-27).

A hypothetical homology model of SmGBP was subsequently generated by using the rat mGluR3 extracellular domain as a structural template. The model suggests that SmGBP has the characteristic bilobed Venus Flytrap structure of the mGluR ligand binding region and shows many conserved glutamate binding residues, suggesting this is a functional binding protein (Fig. 1B). Residues that have been directly implicated in glutamate binding in the rat mGluR3 receptor [38] and are conserved in SmGBP include Arg_68_, Ser_144_, Thr_167_, Tyr_215_ and Asp_297_. (Fig. 1C).

When compared to the general database at NCBI, SmGBP was found to be most closely related to insect, nematode and vertebrate mGluRs (Blastp E values < −84). In addition, SmGBP shares sequence homology with other glutamate receptors of *S. mansoni*, including the previously described SmGluR [25], as well as predicted mGluRs of flatworm species for which genome sequences are available, notably *Schistosoma japonicum* and the planarian *Schmidtea mediterranea*. A search of the *S. japonicum* database available at GeneDB (http://www.genedb.org/) found a protein sequence (Sjp 0088520) of 485 amino acid residues that is almost identical to SmGBP (92% homology) and another highly homologous 486 amino acid sequence was detected in the *S. mediterranea* genome database (v31.015264) (http://smedgd.neuro.utah.edu) [26]. Interestingly, these two flatworm proteins are also C-terminally truncated and lack the same domains (cysteine-rich linker, the heptahelical transmembrane segment and the carboxy intracellular domains) as SmGBP.

**Figure 2:**
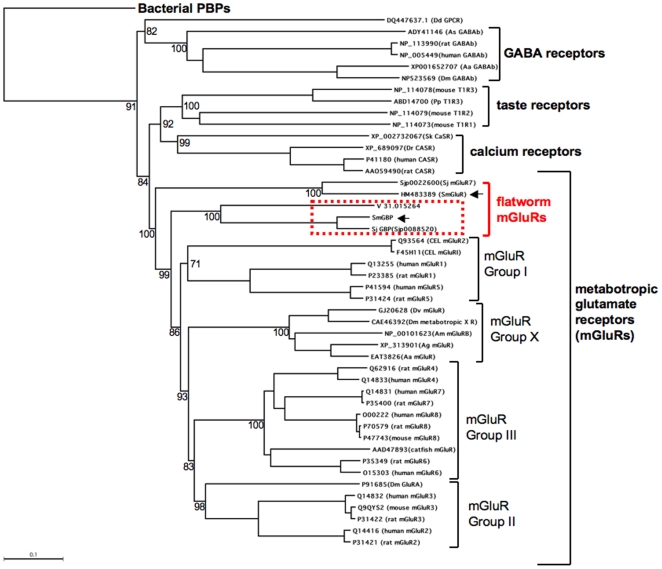
Dendogram analysis showing the relationship of SmGBP with structurally related receptors from *S. mansoni* and other species. The amino acid sequences were aligned using the ClustalW method and a Neighbor-Joining Best Tree was constructed from the multiple sequence alignment. Included in the alignment are representative examples of all major groups of metabotropic glutamate receptors (mGluR Groups I, II, III), an insect-specific group of mGluRs (Group X) and several flatworm mGluR-like sequences. For comparison, we also included other types of metabotropic (Family C) G protein-coupled receptors (calcium-sensing, taste and γ-aminobutyric acid (GABA) receptors) along with bacterial periplasmic binding proteins (PBP) as the outgroup. Five bacterial PBP sequences were included in the alignment (Accession # AAC13287, AAM84023; BAB40635; ZP_03825963; YP_011745770) but they were omitted from the tree for simplicity. The tree was rooted to one of the bacterial PBPs (Accession # YP_011745770) and was tested by bootstrap analysis with 1000 iterations. The length of the branches is proportional to the genetic distance between sequences and bootstrap values (normalized to 100) are provided for selected key branches. Sjp_0022600 (SjmGluR7) and Sjp_0088520 (SjGBP) are predicted protein sequences obtained from the *S. japonicum* genome database [14] available at GeneDB (http://www.genedb.org/); V31.015264 is a prediction from the *Schmidtea mediterranea* genome database (http://smedgd.neuro.utah.edu) [26]. The remaining protein sequences were obtained from the general database available at the National Center for Biotechnology Information (NCBI) and are identified by their corresponding accession numbers. Species abbreviations are as follows: Aa, *Aedes aegypti*; Ag, *Anopheles gambiae*; Am, *Apis mellifera*; As, *Ascaris suum*; CEL, *Caenorhabditis elegans*; Dd, *Dictyostelium discoideum*; Dm, *Drosophila melanogaster*; Dr, *Danio rerio*; Dv, *Drosophila virilis*; Pp, *Pongo pygmaeus*; Sk, *Saccoglossus kowalevskii*; Sm, *Schistosoma mansoni*; Sj, *Schistosoma japonicum*. The truncated flatworm receptors are boxed and the *S. mansoni* sequences are identified by arrowheads. mGluR, metabotropic glutamate receptor; GBP, glutamate binding protein (truncated mGluR).

Phylogenetic trees were constructed from a ClustalW multisequence alignment of mGluRs from various vertebrate and invertebrate (insect, nematode, flatworm) species, along with other related Family C GPCRs (GABA, calcium-sensing and taste receptors) and bacterial periplasmic binding proteins (PBP) as the outgroup (Fig. 2). The analysis shows the different GPCRs clearly separated on the tree. The major groups of mGluRs (Groups I, II, III) [15] and the insect-specific mGluRX receptors [18] are also shown. Compared to these sequences, the flatworm receptors represent the earliest divergence from the mGluR ancestor. The *S. mansoni* SmGluR receptor [25] and a related *S. japonicum* homologue (Sj mGluR7) diverged first and constitute a separate branch in the mGluR tree. SmGluR is only distantly related to vertebrate mGluRs but it was shown to be selectively activated by glutamate *in vitro* [25] and therefore it is considered to be a functional receptor. The remaining flatworm sequences in this alignment are the truncated receptors, SmGBP, *S. japonicum* SjGBP and the planarian homologue (Fig. 2, boxed sequences), which also constitute a separate branch in the tree. These sequences diverged after SmGluR but before the separation of the major groups of glutamate receptors. Thus we conclude that schistosomes have at least two different and novel types of mGluR-like proteins represented by SmGluR, SmGBP and their *S. japonicum* homologues. In addition, there is one more predicted mGluR in *S. mansoni* (Smp_153370) [13] and a related partially annotated *S. japonicum* sequence (Sjp_0094830) [14], which could constitute a third type of receptor (not shown).

### Immunoprecipitation experiments

**Figure 3:**
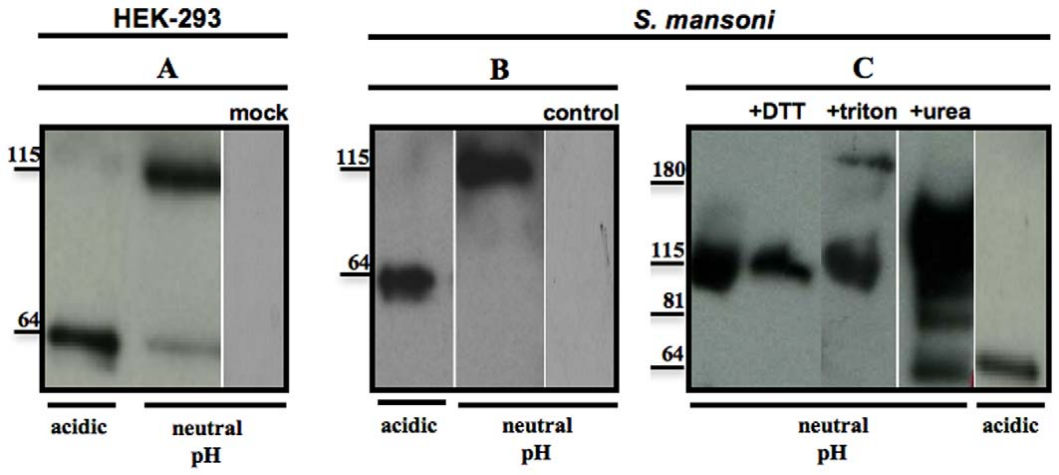
Immunoprecipitation (IP) of recombinant and native SmGBP. Recombinant SmGBP was immunoprecipitated from transfected HEK-293 cell lysates (A) and native SmGBP was immunoprecipitated from a preparation of solubilized *S. mansoni* membrane proteins (B, C), as described in the text. In both cases, the IP was performed with anti-SmGBP antibody that was covalently linked to agarose beads. The protein was eluted from the beads at pH 2.8 (acidic) and the eluate was subsequently neutralized to pH 7.4 (neutral pH). Aliquots of the acidic and neutralized IP eluate were immunoblotted with peptide purified anti-SmGBP antibody. Under acidic conditions the antibody identified one major band at ≈ 60 kDa, whereas upon neutralization the antibody recognized a second band at ≈ 115 kDa both in transfected HEK-293 cells and *S. mansoni*. No bands were seen in the negative controls, either HEK-293 cells transfected with empty plasmid (mock) or blots probed with preimmune serum (control). (C) Native SmGBP was immunoprecipitated as above and treated with 0.1 M DTT, 1% triton X-100, 6 M urea or acid conditions (pH 2.8) prior to western blotting with anti-SmGBP antibody. The positions of relevant molecular weight markers are shown.

To investigate SmGBP at the protein level, we obtained a specific polyclonal antibody targeting two unique SmGBP peptides. The antibody was affinity-purified and tested first against recombinant SmGBP expressed in HEK-293 cells. *In situ* immunofluorescence assays showed that the antibody recognized cells transfected with SmGBP expression plasmid (pCIneo-SmGBP) but not the control cells transfected with empty plasmid (Fig. S1). SmGBP was subsequently immunoprecipitated from transfected HEK-293 cell lysates, using anti-SmGBP antibody that was covalently linked to agarose beads. Proteins were eluted from the antibody-coupled beads under acidic conditions (pH 2.8), neutralized (pH 7.4) and then immunoblotted with affinity-purified anti-SmGBP (Fig. 3A). The results showed two species of SmGBP, one corresponding roughly to the size of the monomer (≈60 kDa) and a larger species of about twice the size at ≈115 kDa. Neither of these bands could be seen in the mock-transfected control and therefore they were considered to be specific. The 60 kDa band was predominant in the initial acidic eluate. However, upon neutralization we saw an increase in the proportion of the 115 kDa species and a corresponding decrease in the intensity of the 60 kDa band (Fig. 3A), suggesting the two species are interconvertible. mGluRs are known to form stable dimers that resist denaturation on SDS-PAGE and can be either covalent or non-covalent in nature [22]-[24], [39], [40]. Our results suggest that the 115 kDa species is probably a non-covalent, pH-sensitive SmGBP dimer that becomes dissociated at acidic pH. To test whether these forms of SmGBP also exist in *S. mansoni*, we performed IP experiments on adult *S. mansoni* extracts. A preliminary western blot analysis determined that SmGBP was enriched in the membrane fraction of *S. mansoni* and therefore we immunoprecipitated SmGBP from purified *S. mansoni* membrane proteins, using the same anti-SmGBP antibody-coupled beads. The IP results are virtually identical to those obtained with the recombinant protein. In both cases, the anti-SmGBP antibody detected a predominant ≈ 115 kDa species at neutral pH and a band of about half the size (≈ 60 kDa) when the sample was acidified (Fig. 3B). Further analysis revealed that the 115 kDa species was resistant to treatment with a reducing agent (0.1M DTT) and detergent (1% triton-x100) but it could be made to dissociate partially upon treatment with 6 M urea and it was completely dissociated at acidic pH, such that only the monomer (≈ 60 kDa) was visible at pH ≈ 3 (Fig. 3C). This supports the notion that the larger SmGBP species is a stable but non-covalent, pH-sensitive dimer. No bands were detected in IP eluates probed either with preimmune serum or a peptide-preadsorbed antibody control (Fig. 3B).

### SmGBP is a membrane-associated protein in *S. mansoni*

**Figure 4:**
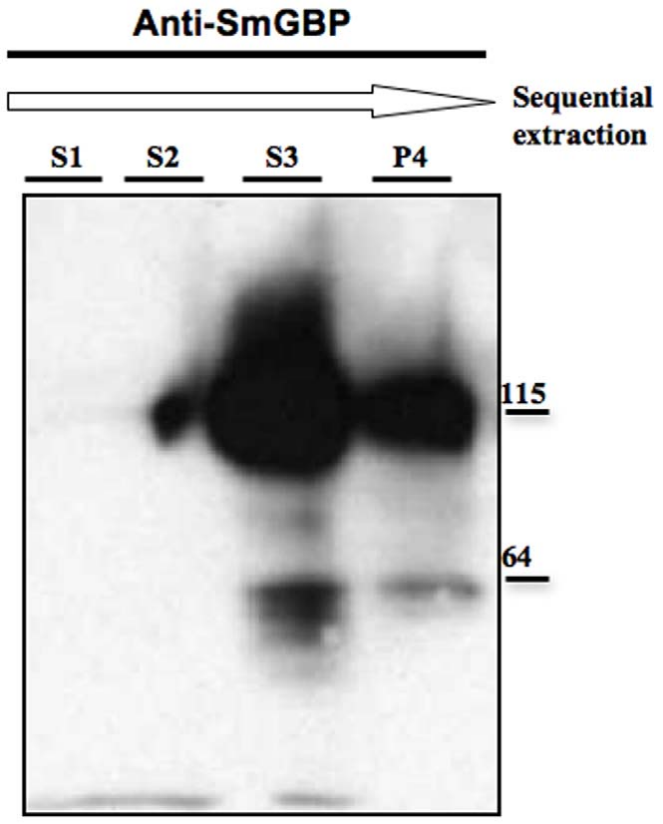
Native SmGBP is a membrane –associated protein. *S. mansoni* peripheral and integral membrane proteins were fractionated by sequential extraction in high salt, alkaline pH and urea, as described in the text. Aliquots of the supernatants after extraction in 2 M NaCl (S1), bicarbonate buffer, pH 11.3 (S2), 4 M urea (S3) and the final pellet (P4) were immunoblotted with peptide-purified anti-SmGBP antibody. The antibody recognized two major bands corresponding to the monomer and predicted dimer in the S3 supernatant after 4 M urea extraction as well as in the final pellet.

Since SmGBP lacks its 7-TM membrane-anchoring segment, we questioned whether this protein was associated with the membrane, as expected of a cell surface receptor, or whether it was expressed as a soluble protein. In preliminary experiments, we isolated membrane proteins from adult *S. mansoni*, using a commercial kit, and then immunoblotted both the membrane fraction and the remaining (predominantly soluble) fraction with the specific anti-SmGBP peptide antibody. The results of this preliminary analysis revealed that the native SmGBP was enriched in the membrane fraction and was associated with the same two major western positive bands (≈ 60 and 115 kDa) described above. To verify these results, we performed sequential extractions of *S. mansoni* proteins and examined each step by western blot analysis (Fig. 4). First, we homogenized adult worms in high-salt solution (2 M NaCl) to release both cytosolic (soluble) and loosely associated peripheral membrane proteins (S1). This was followed by treatment with high pH carbonate buffer, which breaks membrane-bound vesicles and releases their protein content into the supernatant, and where proteins associated peripherally with the membrane can also be found (S2). In the last step, 4 M urea was used to remove tightly associated peripheral proteins from the membrane (S3) and the remaining pellet (P4) was expected to contain predominantly integral membrane proteins [31]. Western blotting with affinity-purified anti-SmGBP antibody revealed that SmGBP was extracted after treatment with urea (S3) but a significant amount was retained in the final pellet, mostly as the larger MW (≈ 115 kDa) species (Fig. 4). Thus we conclude that the native SmGBP is either an integral membrane protein, or a peripheral membrane protein that is tightly associated with the membrane.

### Expression of SmGBP in adult male *S. mansoni*

**Figure 5:**
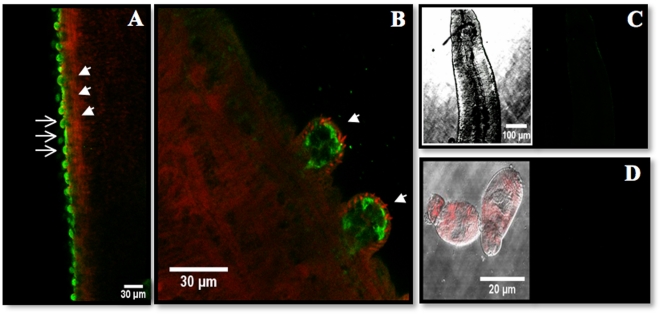
Tissue localization of SmGBP. *S. mansoni* were probed with affinity-purified anti-SmGBP antibody followed by a FITC-labeled secondary antibody. (A) Strong immunoreactivity (green) was detected on the surface layer of adult male worms (open arrows). Actin-rich muscles were stained with TRITC-labeled phalloidin (red) and can be seen beneath the tegument (solid arrow heads). At higher magnification, the SmGBP signal was found to be enriched in the tubercles (arrows) of the male, where numerous actin-rich phalloidin-labeled spines (red) can also be seen (B). No significant SmGBP immunofluorescence was detected in male worms probed with peptide-preadsorbed antibody (C). Besides males, we did not observe specific SmGBP immunoreactivity in any of the other stages tested, including cercariae (D) schistosomula and female worms (not shown).

The tissue localization of SmGBP in adult and larval stages was subsequently examined by confocal immunofluorescence, using affinity-purified anti-SmGBP antibody followed by an FITC-labeled secondary antibody. Analysis of cercariae and schistosomula stages found no detectable SmGBP expression. Likewise, we did not see any immunoreactivity in female worms, apart from inconsistent fluorescence in the vitellaria and intestinal caeca, which could also be detected in some of the controls and was deemed to be non-specific. In contrast, SmGBP was highly expressed in adult male worms. Expression in the males was confined to the surface layer, especially the dorsal tubercles, and was seen along the length of the body (Fig. 5A). Counterstaining with TRITC-conjugated phalloidin revealed the somatic muscles located beneath the tegument but no co-localization of SmGBP (green) with cytoskeletal elements or muscle (red) could be seen. In the tubercles, the green SmGBP fluorescence is surrounded by red actin-rich spines, again with no detectable co-localization (Fig. 5B). No comparable green fluorescence could be seen in any of the controls done, including peptide antigen preadsorbed antibody control (Fig. 5C), omission of primary antibody or pre-immune serum.

### Biotinylation of *S. mansoni* surface proteins

**Figure 6:**
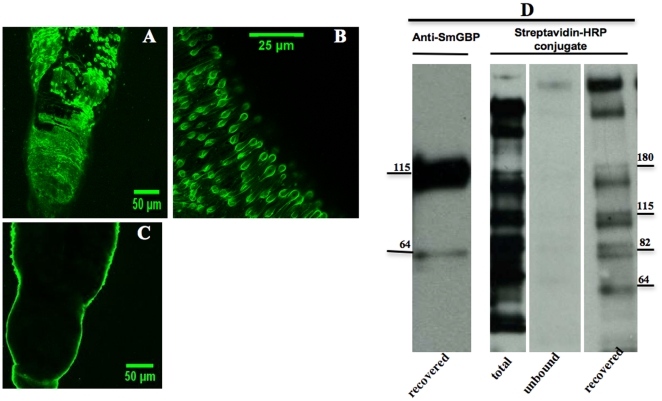
Surface biotinylation of adult *S. mansoni*. Adult male worms were incubated with EZLink™ sulfo-NHS-LC-biotin followed by streptavidin- FITC conjugate. Examination of biotinylated animals by confocal microscopy showed that the labeling was confined to the surface (A, B). No significant penetration of streptavidin-FITC could be seen in the internal tissues or gut (C). Western blot analyses (D) were performed on aliquots of total biotinylated proteins (total), streptavidin-purified proteins (recovered) and the flow-through (unbound). Blots were probed first with streptavidin-conjugated to HRP to verify biotin labeling and recovery of biotinylated proteins. The streptavidin-purified fraction (recovered) was subsequently probed with anti-SmGBP antibody. The relevant molecular weights are indicated.

Biotinylation experiments were done to verify the surface localization of SmGBP. We used a hydrophilic biotinylated reagent (EZ-Link® sulfo-NHS-LC-Biotin) that is excluded by the schistosome membranes and will bind proteins near the surface of an intact worm [33]. The labeled proteins were then recovered by affinity-coupling to streptavidin. Before labeling, the worms were examined carefully under the microscope to check the integrity of the surface membrane and the biotinylation was monitored with streptavidin-FITC conjugate. FITC fluorescence was consistently observed on the surface of the worm, as expected (Fig. 6A, B) but not inside the animal (Fig. 6C). Aliquots of total biotin-labeled membrane protein, unbound and streptavidin-purified proteins were tested in western blotting using streptavidin-horseradish peroxidase (HRP) conjugate to verify biotin labeling. Streptavidin binding was detected in the total biotin-labeled membrane and the recovered fraction, while very little, if any, was seen in the unbound fraction, as expected (Fig. 6D). Streptavidin-recovered protein was subsequently probed with anti-SmGBP antibody to test if the receptor was present in this protein fraction. The antibody recognized the same two bands of the expected size, ≈60 kDa and 115 kDa, of which the largest species is the most abundant.

### Quantitative analysis of SmGBP mRNA expression

**Figure 7:**
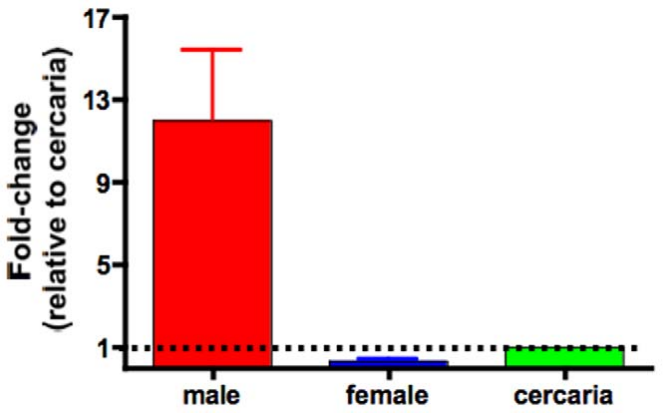
Developmental and gender-specific expression of SmGBP. Quantitative PCR was performed on reverse-transcribed cDNA from *S. mansoni* adult males, females and cercariae. The data were normalized to *S. mansoni* GAPDH, which was used as an internal housekeeping gene. Differences in expression were calculated according to the comparative ΔΔCT method [34]. The results are shown as fold-change in *S. mansoni* SmGBP expression relative to cercariae. Data are the means and SEM of seven independent experiments, each in duplicate.

The confocal immunolocalization experiments suggest that SmGBP is enriched in males and may be absent in females and larval stages. Subsequent western blot analyses of membrane protein extracts from cercariae and 5-day old schistosomula also failed to detect SmGBP (data not shown). To verify these results, we measured expression of SmGBP at the mRNA level by real-time qPCR. Equal amounts of male, female and cercaria RNA were used in a reverse-transcription qPCR experiment, which also included a minus reverse-transcriptase control to detect genomic contamination and a non-template control. Data were normalized relative to a housekeeping gene (GAPDH) and changes in expression level were calculated by the ΔΔ_CT_ method [34], using cercariae as our stage of reference. The results confirmed our earlier observations from the protein studies. The level of SmGBP expression in adult males is ≈ 12 fold higher than that in cercariae (Student's *t*- test: *P* value  =  0.02). Females have barely detectable expression levels, representing approximately 1/3 of the cercarial level (0.338, Student's *t*- test: *P* value  =  0.000135) and nearly 40 times less than the males (Fig. 7).

### Bacterial expression of SmGBP

**Figure 8:**
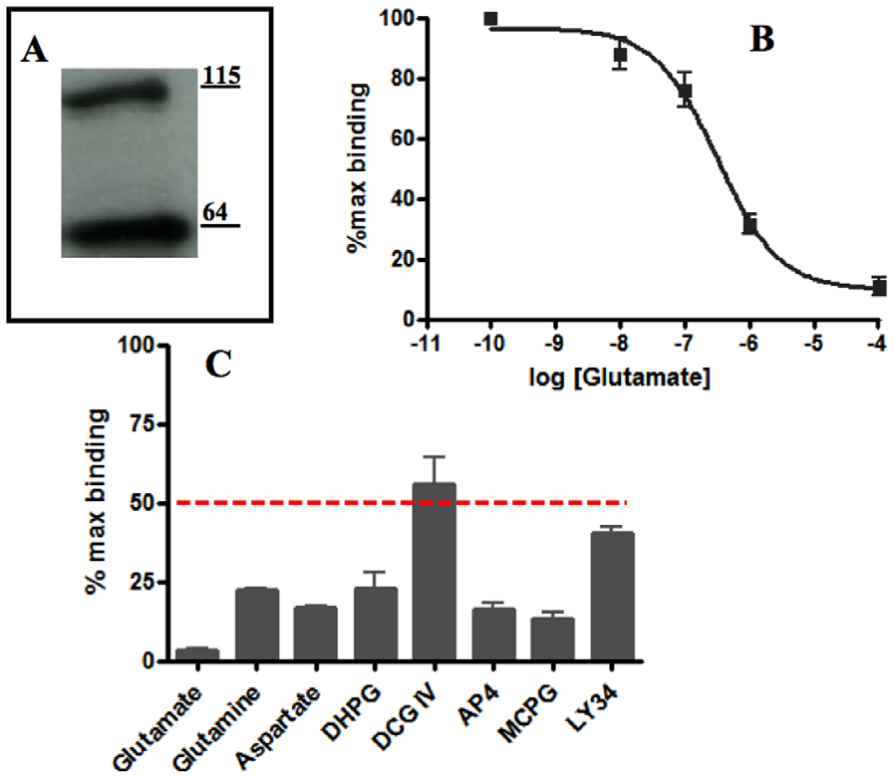
[^3^H] Glutamate binding assay. (A) SmGBP was expressed in *E.coli* (BL21) as a C-terminal oligohistidine fusion protein, purified by affinity chromatography and tested by western blotting with anti-SmGBP antibody. The antibody identified two immunoreactive protein species corresponding to the SmGBP monomer and dimer (B) Purified His-SmGBP was incubated with [^3^H] glutamate and increasing concentrations (10^−10^ to 10^−4^) of unlabelled glutamate for 1 h at 4°C and the bound ligand was recovered by polyethylene glycol (PEG) precipitation. The data were normalized relative to the maximum level of [^3^H] glutamate binding measured in the absence of cold ligand and each point is the mean and SEM of four experiments done in duplicates. (C) Purified His-SmGBP was incubated with a constant amount of [^3^H] Glutamate and an excess of test substance, each added at 10 mM. Experiments were performed using glutamate, glutamine, aspartate and the following glutamatergic ligands: (S)-3,5-DHPG ((S)-3,5-Dihydroxyphenylglycine), DCG IV (2S,2′R,3′R)-2-(2′,3′-Dicarboxycyclopropyl)glycine), L-AP4 (L-(+)-2-Amino-4-phosphonobutyric acid), (*S*)-MCPG ((S)-a-Methyl-4-carboxyphenylglycine), LY 341495 ((2S)-2-Amino-2-[(1S,2S)-2-carboxycycloprop-1-yl]-3-(xan th-9-yl) propanoic acid). Data were normalized relative to the control sample that contained [^3^H] glutamate without competitor. The results are the means and SEM of four separate experiments, each in duplicate. For aspartate and glutamine, the data are derived from two experiments, each in triplicate. All drugs tested were significantly different from the no drug control at *P* <0.05.

The 1452bp coding sequence of SmGBP was amplified by PCR, cloned into the prokaryotic vector pET22b and expressed in *E. coli* BL21(DE3)pLysS as a C-terminal oligo-histidine fusion protein. The pET22b vector is designed to direct the expressed protein to the periplasmic space. Periplasmic expression was selected for this study because SmGBP was found to be toxic to the bacterium when overexpressed in the cytoplasm. The histidine-tagged SmGBP was purified by nickel affinity chromatography and tested first by western blot analysis with anti-SmGBP antibody. The results identified the same two western-positive bands at ≈ 60 kDa and 115 kDa (Fig. 8A). The purified protein was subsequently tested for glutamate binding activity. Aliquots of recombinant His-tagged SmGBP were incubated with a constant amount of [^3^H]glutamate in the presence of increasing concentrations of unlabelled glutamate (10^−10^ -10^−4^ M) and the binding affinity was calculated by non-linear curve fitting of the resulting competition curve (IC_50_ 0.3 ± 0.05 µM) (Fig. 8B). To test whether SmGBP recognizes classical (mammalian) mGluR ligands, we repeated the [^3^H]glutamate competition assays with different glutamatergic agonists and antagonists in addition to glutamine, aspartate and glutamate itself (Fig. 8C). Several of the test drugs were able to compete with [^3^H]glutamate, AP4 (mGluR III agonist) and S)-MCPG (mGluRs I/II antagonist) being the strongest, followed by (S)-3,5-DHPG (mGluRI agonist), whereas DCG IV (mGluR II agonist) and Ly341495 (mGluR II antagonist) were poor competitors (≈50% inhibition). Among the amino acids, glutamate was the strongest competitor, as expected, but we also observed competition by glutamine and aspartate (77% and 84% inhibition of [^3^H] glutamate binding, respectively), suggesting that SmGBP can also recognize these amino acids.

## Discussion

L-Glutamate is a major excitatory neurotransmitter in the CNS of vertebrates and many invertebrate phyla but its role in schistosomes is poorly understood. Here we describe one of at least three mGluR-like sequences encoded in the *S. mansoni* genome [13]. SmGBP is an unusual receptor; it is a C-terminally truncated mGluR that has a conserved glutamate binding site but lacks the heptahelical and intracellular domains. The predicted protein sequence was deduced from the genomic DNA sequence, which is similarly truncated, and was verified experimentally by RACE and cloning of the cDNA. Shorter variants of mGluRs have been described in mammalian species but these are produced from alternative splicing of longer transcripts and usually retain the 7-TM membrane anchoring region. The majority of mGluR splicing events that have been reported to date occur within the intracellular C-terminal tail and do not involve the transmembrane domains [41]. In a few instances, however, researchers have reported unusually short splice variants that lack the entire 7-TM region and are similar in length to SmGBP. The best example is the human mGluR3 receptor, which exists both as a full-length GPCR and a C-terminally truncated variant of unknown function that carries only the ECD [42]. We considered the possibility that SmGBP might also be encoded by a longer gene sequence that has yet to be annotated. However, we could not identify any splice variants of SmGBP using RACE techniques. More than ten 3′ RACE clones were examined and all were found to be similarly truncated. In addition, the SmGBP cDNA was found to be polyadenylated, suggesting that the transcript is mature and properly processed in the worm. Thus we conclude that SmGBP is expressed, at least in part, as a short receptor. We cannot, however, rule out the possibility of a longer variant of this protein that could not be detected in this study.

The ECD of eukaryotic mGluRs is distantly related to periplasmic amino acid binding proteins (PBPs) normally seen in bacteria. Bacterial PBPs constitute a large family of proteins that mediate transmembrane transport of a variety of nutrients, including amino acids, and they also play an important role in signaling, for example in chemotaxis [43]. They lack transmembrane domains and are expressed either as soluble proteins in the periplasmic space of gram-negative bacteria, or are attached to the cytoplasmic membrane via a lipid anchor in gram-positive bacteria [44]. Amino acid- binding PBPs share significant structural homology with the Venus Flytrap module of mGluRs and are believed to have a common origin. It has been suggested that glutamate receptors have evolved from the fusion of a bacterial PBP gene with a gene encoding the membrane anchoring region, either a channel in the case of ionotropic receptors, or a heptahelical 7-TM receptor in the case of mGluRs [21], [45]. This raises interesting questions about the evolution of the truncated schistosome receptor. One possibility is that SmGBP might be related to an ancestor of mGluRs that existed before the gene fusion event but the phylogeny makes this seem unlikely. Our sequence analyses show that SmGBP is more closely related to mGluRs than to bacterial PBPs. Moreover the other schistosome glutamate receptor (SmGluR) is a full-length protein that comprises both the ligand binding domain and the 7-TM region [25], indicating that modular mGluRs already existed in the ancestor of flatworms. An alternative, more plausible explanation is that SmGBP evolved from a full-length eukaryotic receptor but subsequently lost its C-terminal half, perhaps by introduction of splicing signals that over time resulted in the loss of 3′ end exons. We have found truncated sequences similar to SmGBP in *S. japonicum* and the planarian, *S. mediterranea*, but not in vertebrates or other invertebrates. Thus SmGBP is conserved within the phylum and could be a flatworm-specific adaptation.

To further characterize this protein, we obtained a polyclonal antibody against two SmGBP-specific peptides. Western blot analyses of proteins from *S. mansoni* and HEK-293 cells expressing SmGBP detected a band at ≈60 kDa and a second band of about 115 kDa in both systems. These bands were not present in the mock-transfected cells or any of the antibody controls, which indicates the specificity of the two protein forms. To verify these results we immunoprecipitated SmGBP from both *S. mansoni* and transfected HEK-293 cells, using the same antibody, and then the purified proteins were tested by western blotting. Once again, two major SmGPR species of about 60 kDa and 115 kDa were consistently observed. The 60 kDa corresponds roughly to the expected size of the SmGBP monomer (55 kDa). The small difference could be due to glycosylation at any one of 4 possible N-glycosylation sites (positions 81, 202, 308 and 479) and also the high pI of the protein (pI  =  9.0), which is known to cause aberrant migration on SDS-PAGE. The second, larger immunoreactive band of 115 kDa is likely a SmGBP dimer that resists denaturation on SDS-PAGE gels. SDS-resistant dimers are common for G protein-coupled receptors and recent work has shown that most family C GPCRs, including mGluRs are obligate dimers [22], [39], [40]. Dimerization is necessary for receptor activation; glutamate binding to one ECD causes only partial activation, and binding to both domains is required for full activation [23], [24]. Several structural mechanisms are involved in mGluR dimerization. Early studies identified covalent interactions due to disulfide bonds located within the N-terminus [39]. However non-covalent interactions involving the extracellular region have also been proposed [46]. The dimer of SmGBP is most probably formed by non-covalent interactions since it was found to be resistant to a reducing agent (DTT) but it could be made to dissociate when treated with agents that disrupt non-covalent bonds, such as urea and low pH.

To test for binding activity, SmGPB was expressed in *E. coli* as an oligohistidine-tagged protein and purified by metal-chelation chromatography. The purified SmGBP was shown to bind glutamate specifically and in a dose-dependent manner, and also recognized some glutamatergic agents, for example, agonists DHPG and AP4 and the antagonist, MCPG. Glutamine and aspartate were both competitors of [^3^H] glutamate but not as potent as unlabelled glutamate itself. The results show that SmGBP is a functional glutamate-binding protein. This is consistent with earlier studies of truncated forms of recombinant mGluRs. The ECDs of mGluR1, mGluR4 and mGluR8 were all expressed heterologously as soluble proteins and were shown to retain their ligand binding activity [47], [48].

SmGBP lacks the heptahelical membrane domain and therefore it was expected to be a soluble protein. Surprisingly, however, we found the native protein to be enriched in the membrane fraction of *S. mansoni*. Moreover, our biochemical analysis suggests that the association with the membrane is strong, consistent with that of a tightly-bound peripheral or integral membrane protein. These results are similar to a recent investigation of the truncated splice variant of the mammalian mGluR3 receptor, which also lacks its 7-TM segment and yet was found to be associated with the plasma membrane [42]. In this case, the authors identified a short hydrophobic segment located at the C-terminal end that was predicted to serve as a single transmembrane domain [42]. The sequence analysis of SmGBP detected a putative TM domain at the N-terminal end. It is possible that SmGBP is inserted into the membrane via this N-terminal TM region. Alternatively, SmGBP may attach to the membrane by non-covalent interactions with a membrane component, for example another protein, or it may be covalently linked to a membrane lipid, similar to the PBP-like proteins of gram-positive bacteria [44]. The sequence analysis of SmGBP did not show high probability of covalently bound lipid anchors (palmitoylation, myristoylation or glycosylphosphatidylinositol (GPI)) but this possibility cannot be ruled out.

To elucidate the function of SmGBP in *S. mansoni*, we examined the tissue localization using a specific antibody. Previously, glutamate was detected in the nervous system of *S. mansoni* [9] and glutamate agonists were reported to have an effect on motor activity [12]. Thus we had expected to find SmGBP in the CNS or the peripheral innervation of the musculature but, surprisingly, no expression was detected in these sites. Instead, SmGBP localized to the surface layer of male worms, especially the tubercles, and there was no visible association with the subtegumental muscles in phalloidin-labeled animals. Subsequent biotinylation experiments detected significant SmGBP immunoreactivity in the steptavidin-purified biotinylated fraction, again suggesting that the protein is located at or near the parasite surface. Interestingly, we could not detect SmGBP protein either in female worms or larval stages, including the free-living larvae (cercariae) or schistosomula. This selective expression in males was also observed at the RNA level. The qPCR experiments showed that SmGBP was upregulated several-fold in adult males compared to cercariae and it was barely detectable in female worms. A previous microarray transcriptome analysis of *S. mansoni* identified SmGBP (Smp_052660) as one of the genes showing differential expression between male and female worms [49]. Our results support this earlier analysis.

The tissue localization of SmGBP offers possible clues about its function in schistosomes. The absence of the protein in neuromuscular structures suggests this is probably not the receptor responsible for the previously described motor effects of glutamate; rather the data suggest that SmGBP is involved in some other form of glutamate signaling that occurs at the parasite surface. The tubercles, where SmGBP is enriched, are protuberances of the male dorsal surface. Several neurotransmitters and neuronal proteins have been identified in the tubercles of schistosomes, where they are believed to be associated with sensory nerve endings [28], [50]–[53]. The pattern of SmGBP immunoreactivity resembles that of other neuronal proteins in these regions, suggesting the receptor could be expressed in nerve endings along the surface of male worms and could play an important role in chemosensory signaling, either as part of an endogenous pathway or in response to exogenous (host-derived) glutamate.

Although SmGBP can bind glutamate, it lacks the mid- and C-terminal regions required for signaling and therefore it is unlikely to have activity on its own. Its effects are probably mediated by interactions with other proteins that are present in the tubercles. Efforts to identify SmGBP-interacting partners using co-immunoprecipitation and proteomics methods have not been successful thus far (data not shown) and therefore the identity of these proteins remains unclear. One possibility is that SmGBP binds to another glutamate receptor to modulate signaling activity. In mammals there is evidence that a truncated mGluR mutant can dimerize with the full-length receptor and the interaction is dominant negative, resulting in decreased signaling. This effect is believed to represent an important mechanism of mGluR regulation in mammalian cells [54] and it is possible that SmGBP functions in a similar manner in *S. mansoni*. SmGBP is unlikely to interact with the previously described SmGluR schistosome receptor because their tissue distributions are quite different [25] but there is at least one more predicted mGluR in the genome whose distribution is unknown [13] and there could be other potential partners that have yet to be identified.

SmGBP may have functions that are unrelated to neuronal signaling. The location of the protein so close to the surface raises the possibility that some of the receptor is associated with the male tegument. The tegument is a specialized syncytial epithelium that covers the entire length of the worm, including the tubercles, and plays a variety of key roles, notably in nutrient transport, immune evasion and interactions with the host [55]. A tegumental glutamate receptor could be used to mediate host-parasite communication, as suggested for a number of other receptors that have been localized to the schistosome tegument [28], [53], [56]. In addition, given the prevalence of SmGBP in males, we note that many of the genes that are upregulated in male schistosomes are involved in macromolecule transport and overall energy production [57]. SmGBP could interact with a tegumental transporter to mediate glutamate intake from the host, a function similar to that of PBPs in bacteria, or it could bind to a metabolic tegumental enzyme that requires glutamate for activity.

In summary, we have identified a novel glutamate receptor that diverged early in evolution and lacks several of the conserved domains normally seen in eukaryotic mGluRs. The unusual structure of this protein, combined with its surface location make SmGBP an attractive target for drug or vaccine intervention. More research is needed to elucidate the functional role of this receptor in the parasite.

## Supporting Information

Figure S1 Heterologous expression of *S. mansoni* SmGBP in cultured mammalian cells. HEK293 cells were transiently transfected with expression plasmid pCIneo-SmGBP (panels B, C) or empty plasmid as a control (panel A). Cells were fixed and permeabilized with ice-cold methanol, as described [28], [32] and were subsequently incubated with affinity-purified anti-SmGBP antibody, followed by a secondary Fluorescein Isocyanate (FITC)-conjugated antibody. Cells were counterstained with 4′,6-diamidino-2-phenylindole (DAPI) and examined by confocal microscopy. FITC fluorescence (green), DAPI fluorescence (blue) and the overlay of the two signals are shown for each of the three panels. The results show strong FITC immunoreactivity in cells expressing SmGBP but not the mock transfected control.
(TIF)
