# Supplementary figures and images for: Characterization of a Truncated Metabotropic Glutamate Receptor in a Primitive Metazoan, the Parasitic Flatworm Schistosoma mansoni
Source: PLoS One. 2011 Nov 1;6(11):e27119. doi: 10.1371/journal.pone.0027119 (PMC3206071; doi:10.1371/journal.pone.0027119)

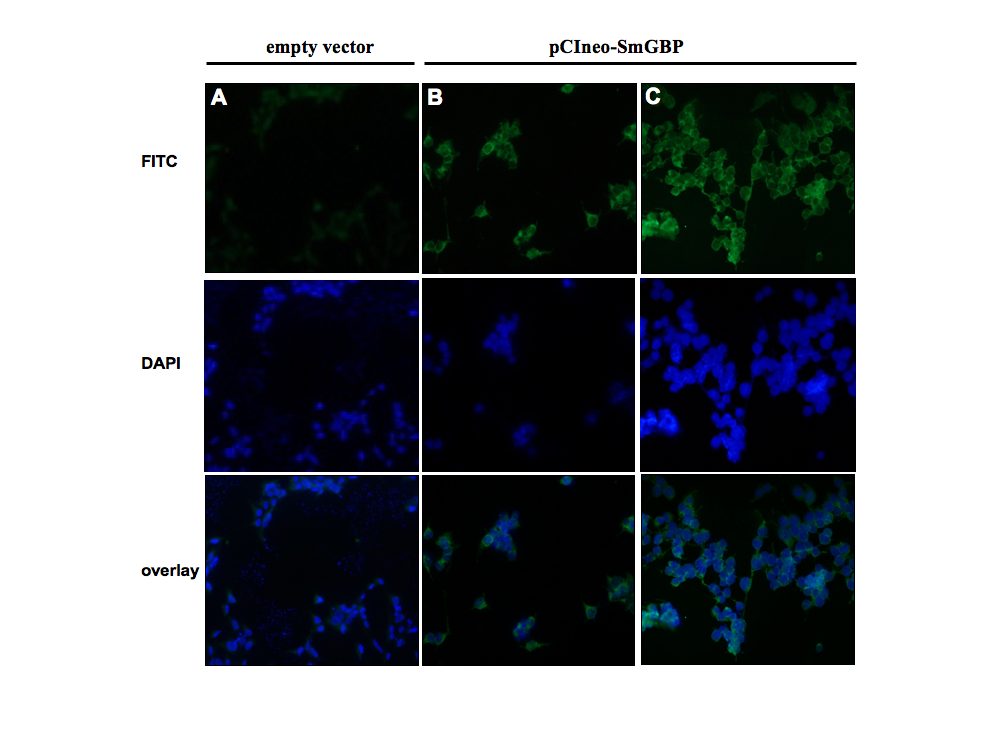

Supplement: Figure S1 — Heterologous expression of S. mansoni SmGBP in cultured mammalian cells. HEK293 cells were transiently transfected with expression plasmid pCIneo-SmGBP (panels B, C) or empty plasmid as a control (panel A). Cells were fixed and permeabilized with ice-cold methanol, as described [28], [32] and were subsequently incubated with affinity-purified anti-SmGBP antibody, followed by a secondary Fluorescein Isocyanate (FITC)-conjugated antibody. Cells were counterstained with 4′,6-diamidino-2-phenylindole (DAPI) and examined by confocal microscopy. FITC fluorescence (green), DAPI fluorescence (blue) and the overlay of the two signals are shown for each of the three panels. The results show strong FITC immunoreactivity in cells expressing SmGBP but not the mock transfected control. (TIF) [file pone.0027119.s001.tif]
